# Prognostic Prediction of Cardiopulmonary Exercise Test Parameters in Heart Failure Patients with Atrial Fibrillation

**DOI:** 10.36660/abc.20180193

**Published:** 2020-02

**Authors:** António Valentim Gonçalves, Tiago Pereira-da-Silva, Rui Soares, Joana Feliciano, Rita Ilhão Moreira, Pedro Rio, Ana Abreu, Rui Cruz Ferreira

**Affiliations:** 1 Centro Hospitalar Universitário Lisboa Central, Hospital de Santa Marta, Lisbon - Portugal

**Keywords:** Atrial Fibrillation/mortality, Peak Expiratory Flow Rate, Exercise Test, Oxygen Consumption, Heart Failure, Prognosis

## Abstract

**Background:**

Atrial fibrillation (AF) is associated with increased mortality in heart failure (HF) patients.

**Objective:**

To evaluate whether the risk of AF patients can be precisely stratified by relation with cardiopulmonary exercise test (CPET) cut-offs for heart transplantation (HT) selection.

**Methods:**

Prospective evaluation of 274 consecutive HF patients with left ventricular ejection fraction ≤ 40%. The primary endpoint was a composite of cardiac death or urgent HT in 1-year follow-up. The primary endpoint was analysed by several CPET parameters for the highest area under the curve and for positive (PPV) and negative predictive value (NPV) in AF and sinus rhythm (SR) patients to detect if the current cut-offs for HT selection can precisely stratify the AF group. Statistical differences with a p-value <0.05 were considered significant.

**Results:**

There were 51 patients in the AF group and 223 in the SR group. The primary outcome was higher in the AF group (17.6% vs 8.1%, p = 0.038). The cut-off value of pVO_2_ for HT selection showed a PPV of 100% and an NPV of 95.5% for the primary outcome in the AF group, with a PPV of 38.5% and an NPV of 94.3% in the SR group. The cut-off value of VE/VCO_2_ slope showed lower values of PPV (33.3%) and similar NPV (92.3%) to pVO_2_ results in the AF group.

**Conclusion:**

Despite the fact that AF carries a worse prognosis for HF patients, the current cut-off of pVO_2_ for HT selection can precisely stratify this high-risk group.

## Introduction

Heart failure (HF) and atrial fibrillation (AF) often coexist,^1^ with AF occurring in some reports in more than 50% of HF patients, and HF in more than one-third of AF patients.^2^ Since the burden of each is growing, they have been called the two new epidemics of cardiovascular (CV) disease.^3^

The presence of AF in HF patients is associated with adverse hemodynamic consequences, which may exacerbate HF, increasing morbidity and mortality.^4-6^

The cardiopulmonary exercise test (CPET) is a powerful predictor of mortality in HF patients and is used as the criterion standard for the need for heart transplantation (HT),^7^ with peak O_2_ consumption (pVO_2_) and the relation between ventilation and CO_2_ production (VE/VCO_2_ slope) as the most used risk assessment tools.^8^ However, less information is known about whether HF patients with AF can be precisely stratified with the current CPET cut-offs for HT selection. Since the combination of HF and AF provide a worse prognosis, a timely referral for HT or mechanical circulatory support could be extraordinarily important to reduce the negative prognostic effect of AF in HF patients.

The present study seeks to compare the prognostic importance in HF patients of CPET parameters in AF versus sinus rhythm (SR) patients.

## Methods

The investigation conforms to the principles outlined in the Declaration of Helsinki. The institutional ethics committee approved the study protocol. All patients provided written informed consent.

### Patient population and study protocol

The study included a single centre analysis of 274 consecutive HF patients referred to our institution with left ventricular ejection fraction (LVEF) ≤ 40% and New York Heart Association (NYHA) class II or III, from 2009 to 2016. All the patients were referred for evaluation with HF team and possible indication for HT or mechanical circulatory support. Patients with elective HT during the follow-up period (patients who had indication for HT and a heart become available in the first year of follow-up) were excluded from the analysis.

Prospective follow-up included initial evaluation within a period of one month in each patient with:

- Clinical data including etiology of HF, implanted devices, medication, comorbidities, NYHA class and Heart Failure Survival Score (HFSS);^9^
- Laboratory data;
- Electrocardiographic data;
- Echocardiographic data;
- CPET data.

Patients were excluded if one of the following:

- Age < 18 years;
- Planned percutaneous coronary revascularization or cardiac surgery;
- Elective HT in the follow-up period;
- Exercise-limiting comorbidities (cerebrovascular disease, musculoskeletal impairment, or severe peripheral vascular disease);
- Previous HT.

### Follow-up and endpoint

All patients were followed-up for 12 months from the date of completion of the aforementioned complementary exams.

The primary endpoint was a composite of cardiac death or urgent HT (occurring during an unplanned hospitalization with dependency of inotropes for worsening HF). Data were obtained from the outpatient clinic visits and medical charts review and was complemented with a standardized telephone interview to all patients at 12 months of follow-up. Secondary endpoints included all-cause mortality, sudden cardiac death and death for worsening HF.

### Definition of atrial fibrillation

Only persistent or permanent AF was considered for the analysis. The diagnosis was made by electrocardiographic recording in the initial evaluation.

### Cardiopulmonary exercise testing

A maximal symptom-limited treadmill CPET was performed using the modified Bruce protocol (GE Marquette Series 2000 treadmill). Tha gas analysis was preceded by the calibration of the equipment. Minute ventilation, oxygen uptake and carbon dioxide production were acquired breath-by-breath, using a SensorMedics Vmax 229 gas analyser. The pVO_2_ was defined as the highest 30-second average achieved during exercise and was normalized for body mass.^10^ The anaerobic threshold was determined by combining the standard methods (V-slope preferentially and ventilatory equivalents). The VE/VCO_2_ slope was calculated by least-squares linear regression, using data acquired throughout the whole exercise. Several composite parameters of CPET were also calculated. Patients were encouraged to perform exercise until the respiratory exchange ratio (RER) was ≥1.10.

### Statistical analysis

All analyses compare AF patients with SR patients. Data were analysed using the software Statistical Package for the Social Science for Windows, version 24.0 (SPSS Inc, Chicago IL).

Baseline characteristics were summarized as frequencies (percentages) for categorical variables, as means and standard deviations for continuous variables when normality was verified and as median and interquartile range when normality was not verified by the Kolmogorov-Smirnov test. The Student’s t-test for independent samples or the Mann-Whitney test when normality was not verified were used for the analysis of the variables.

Univariable and multivariable Cox proportional-hazards models were applied, with p values for time-to-event analyses being based on log-rank tests, and hazard ratios for treatment effects and 95% confidence intervals presented to study the combined endpoint considering the follow-up time of 12 months.

For selecting patients who would benefit from early selection for HT or mechanical circulatory support, the primary endpoint was analysed by several CPET parameters for the highest area under the curve (AUC) in the 12 months’ follow-up. Hanley & McNeil test was used to compare two correlated receiver operating characteristics curves.^11^

The guideline recommended cut-off value of pVO_2_ (pVO_2_ ≤ 12 ml/kg/min or ≤ 14 ml/kg/min without beta-blockers (BB)) and VE/VCO_2_ slope (VE/VCO_2_ slope > 35 with a RER < 1.05) for HT^7^ selection were analysed (and compared for positive and negative predictive value (PPV and NPV, respectively) in our population of AF and SR patients.

Statistical differences with a p-value < 0.05 were considered significant.

## Results

### Overview of AF and SR groups

A total of 274 patients were enrolled in the study, with 51 patients in the AF group and 223 in the SR group. The baseline characteristics of SR and AF groups are presented and compared in Table 1.

**Table 1:** Baseline characteristics of AF and SR groups

	SR - n = 223	AF - n = 51	p for ≠ between groups
**Clinical data - characteristics**			
Age	52.61 ± 12.53	57.96 ± 8.61	< 0.001
Female (%)	61 (27.4%)	6 (11.8%)	0.019
BMI^1^ (kg/m^2^)	26.80 ± 4.07	27.47 ± 4.78	0.361
Ischemic etiology (%)	90 (40.4%)	14 (27.5%)	0.087
ACEi^2^/ARA^3^ (%)	211 (96.3%)	50 (98.0%)	0.544
BB^4^ (%)	179 (80.3%)	40 (78.4%)	0.768
MRA^5^ (%)	184 (72.2%)	38 (74.5%)	0.677
Diabetes (%)	43 (21.4%)	10 (22.7%)	0.846
Baseline^6^ ICD (%)	109 (49.8%)	27 (52.9%)	0.493
Baseline^7^ CRT (%)	48 (21.5%)	12 (23.5%)	0.781
HFSS^8^	8.77 ± 0.95	8.22 ± 0.93	< 0.001
**Laboratorial data**			
Glomerular filtration rate (ml/min)	76.84 ± 30.20	65.03 ± 29.05	0.012
Sodium (mEq/L)	137.8 (135.7-139.3)	136.9 (133.6-139.3)	0.052
NT-proBNP (pg/ml)	2,046.79 ± 2,223.07	3,247.38 ± 4,578.571	0.097
**Echocardiographic data**			
LVEDD^9^ (mm/m^2^)	38 (35-43)	38 (35-43)	0.237
LVEF^10^ (%)	29 (22-34)	26 (20-30)	0.010
MR III-IV^11^ (%)	87 (39.0%)	12 (23.5%)	0.073
RV dysfunction (%)	29 (13.0%)	22 (40%)	< 0.001
**CPET data**			
Initial HR^13^	82 (72-92)	83 (70-100)	0.232
Maximal HR	137 (121-157)	130 (115-179)	0.747
Maximal HR predicted (%)	82.77 ± 12.86	86.88 ± 23.37	0.230
Delta HR during exercise	53 (39-71)	52 (34-64)	0.636
HHR1^14^	17 (12-26)	16 (10-25)	0.624
Initial SBP^15^	115 (110-125)	1,110 (100-120)	0.026
Maximal SBP	155.30 ± 26.83	145.92 ± 28.98	0.028
Duration of CPET^16^ (min)	10.83 ± 3.99	8.53 ± 4.30	< 0.001
Peak RER^17^	1.10 ± 0.09	1.11 ± 0.09	0.340
pVO_2_ (ml/kg/min)	20.27 ± 5.54	17.81 ± 5.55	0.005
pVO_2_ predicted (%)	68.12 ± 17.65	63.12 ± 18.29	0.072
VE/VCO_2_ slope	30.64 ± 6.78	34.33 ± 8.88	0.006
OUES	1.83 ± 0.58	1.64 ± 0.60	0.035
AT^18^ time (minutes)	7.49 ± 3.44	5.49 ± 3.63	< 0.001
pVO_2_ (ml/kg/min) at AT	16.35 ± 4.29	14.29 ± 4.32	0.002

Values are mean ± standard deviation or median (interquartile range); p values are calculated by Student´s T-test for independent samples or Mann-Whitney U test as appropriate. SR: sinus rhythm; AF: atrial fibrillation; BMI: body mass index; ACEi: angiotensin-converting enzyme inhibitors; ARA: angiotensin receptor blockers; BB: beta-blockers; MRA: mineralocorticoid receptor antagonists; ICD: implantable cardioverter-defibrillator; CRT: cardiac resynchronization therapy; HFSS: Heart Failure Survival Score; LVEDD: left ventricular end-diastolic diameter; LVEF: left ventricular ejection fraction; MR: mitral regurgitation; RV: right ventricular; HR: heart rate; HRR1: heart rate recovery in the first minute after finishing CPET; SBP: systolic blood pressure; CPET: cardiopulmonary exercise test; RER: respiratory exchange ratio; AT: anaerobic threshold.

In regard to clinical data, AF patients were older (57.96 ± 8.61 vs 52.61 ± 12.53, p < 0.001) and had a lower percentage of females. Medication with angiotensin-converting enzyme inhibitors, angiotensin receptor blockers, BB and mineralocorticoid receptor antagonists were similar and highly prevalent in both groups, and no differences were found regarding implantable cardioverter-defibrillator and cardiac resynchronization therapy between the two groups. There were no significant differences for sodium and NT-proBNP, but glomerular filtration rate (GFR) values were lower in the AF group (65.03 ± 29.05 vs 76.84 ± 30.20, p = 0.012).

Higher percentage of right ventricular dysfunction (40.0% vs 13.0%, p < 0.001) and lower values of LVEF (24.96 ± 7.44 vs 27.91 ± 7.23, p = 0.010), revealed a worse biventricular function in AF group.

CPET data showed no differences regarding heart rate parameters, but the AF group had lower baseline and maximal systolic blood pressure (SBP). Significant differences between the two groups were also observed with prognostic measures of CPET, with a worse status in AF group revealed by a lower CPET duration, pVO_2_, oxygen uptake efficiency slope (OUES), time to anaerobic threshold (AT), pVO_2_ at AT and a higher VE/VCO_2_ slope (Table 1).

### Primary and secondary endpoints

At 1 year, the primary endpoint (cardiac death or urgent HT) had occurred in 27 (9.9%) patients as represented in Table 2. There were no patients requiring mechanical circulatory support**.** The AF group had more events regarding the combined endpoint (17.6% vs 8.1%, p = 0.038), with cardiac mortality alone showing a trend for a worse prognosis in the AF group (11.8% vs 5.4%, p = 0.097), with no statistical difference regarding urgent HT (5.9% vs 2.7%, p = 0.249).

**Table 2:** Adverse events at 12 months follow-up

Adverse events at 12 months follow-up	SR - n (%)	AF - n (%)	p
Combined endpoint	18 (8.1%)	9 (17.6%)	0.038
Total mortality	14 (6.3%)	9 (17.6%)	0.008
Cardiac mortality	12 (5.4%)	6 (11.8%)	0.097
Sudden cardiac death	5 (2.2%)	4 (7.8%)	0.043
Death for worsening HF	7 (3.1%)	2 (3.9%)	0.777
Urgent HT	6 (2.7%)	3 (5.9%)	0.249
Mechanical circulatory support	0 (0%)	0 (0%)	1.000

AF: atrial fibrillation; HF: heart failure: HT: transplantation; SR: sinus rhythm.

Secondary endpoints showed higher all-cause mortality (17.6% vs 6.3%, p = 0.008) and a higher sudden cardiac death (7.8% vs 2.2%, p = 0.043) in the AF group, with no difference regarding death for worsening HF (3.9% vs 3.1%, p = 0.777).

Complete data of univariable Cox analysis for prediction of the primary endpoint is presented in Table 3 and Table 4.

**Table 3:** Univariate Cox proportional-hazards analysis (non-CPET parameters)

Characteristics	All				SR				AF			
	Wald	Hazard ratio	95% CI	p	Wald	Hazard ratio	95% CI	p	Wald	Hazard ratio	95% CI	p
Age	0.092	0.995	0.965-1.026	0.762	0.768	0.984	0.950-1.020	0.381	0.057	1.010	0.933-1.093	0.811
Gender	0.524	0.699	0.265-1.845	0.469	1.041	0.525	0.152-1.812	0.308	1.188	2.397	0.498-11.547	0.276
BMI	1.175	0.947	0.859-1.045	0.278	0.183	0.974	0.863-1.099	0.669	1.906	0.887	0.748-1.052	0.167
Beta-Blocker	5.139	2.469	1.130-5.393	0.023	4.259	2.713	1.051-6.998	0.039	0.877	1.941	0.484-7.779	0.349
Diabetes	0.130	1.197	0.451-3.174	0.718	0.027	0.910	0.297-2.792	0.869	0.691	2.416	0.302-19.326	0.406
Baseline CRT	1.614	1.995	0.687-5.790	0.204	1.047	2.160	0.494-9.446	0.306	1.807	2.940	0.610-14.167	0.179
HFSS	34.893	0.233	0.144-0.378	< 0.001	22.674	0.233	0.128-0.424	< 0.001	8.600	0.243	0.095-0.626	0.003
Glomerular filtration rate	3.520	0.586	0.971-1.101	0.061	2.578	0.985	0.967-1.003	0.108	0.205	0.994	0.969-1.020	0.650
Sodium	27.303	0.787	0.720-0.861	< 0.001	14.635	0.766	0.668-0.878	< 0.001	7.668	0.839	0.726-0.947	0.006
NT-proBNP	20.456	8.212	2.234-12.367	< 0.001	15.171	6.263	1.894-10.223	< 0.001	3.187	2.335	1.285-4.534	0.004
LVEDD	5.670	1.072	1.012-1.135	0.017	3.001	1.077	0.990-1.171	0.083	1.443	1.049	0.970-1.135	0.230
LVEF	18.934	0.887	0.840-0.936	< 0.001	13.810	0.884	0.828-0.943	< 0.001	3.351	0.912	0.826-0.998	0.049
RV dysfunction	21.377	3.758	2.144-6.588	< 0.001	6.160	2.846	1.246-6.499	0.013	8.346	4.267	1.594-11.419	0.004

SR: sinus rhythm; AF: atrial fibrillation; CI: confidence interval; BMI: body mass index; CRT: cardiac resynchronization therapy; HFSS: Heart Failure Survival Score; LVEDD: left ventricular end-diastolic diameter; LVEF: left ventricular ejection fraction; RV: right ventricular.

**Table 4:** Univariate Cox proportional-hazards analysis (CPET parameters)

Characteristics	All				SR				AF			
	Wald	Hazard ratio	95% CI	p	Wald	Hazard ratio	95% CI	p	Wald	Hazard ratio	95% CI	p
Initial HR	0.220	1.006	0.983-1.029	0.639	2.265	1.024	0.993-1.056	0.132	1.414	0.977	0.940-1.015	0.234
Maximal HR	6.259	0.982	0.967-0.996	0.012	0.644	0.992	0.974-1.011	0.422	5.706	0.973	0.951-0.955	0.017
Maximal HR(%)predicted	8.343	0.962	0.937-0.968	0.004	1.864	0.975	0.941-1.011	0.172	5.590	0.958	0.924-0.993	0.018
Delta HR during exercise	10.141	0.969	0.951-0.988	0.001	3.324	0.979	0.956-1.002	0.068	6.527	0.960	0.930-0.991	0.011
HHR1	22.484	0.837	0.778-0.901	< 0.001	15.623	0.829	0.755-0.910	< 0.001	5.939	0.869	0.777-0.973	0.015
Initial SBP	13.913	0.946	0.919-0.974	< 0.001	8.317	0.951	0.919-0.984	0.004	4.346	0.939	0.885-0.996	0.037
Maximal SBP	21.896	0.959	0.943-0.976	< 0.001	12.029	0.964	0.945-0.984	0.001	7.205	0.954	0.922-0.987	0.007
Duration of CPET (min)	26.781	0.756	0.681-0.841	< 0.001	20.636	0.730	0.637-0.836	< 0.001	4.009	0.838	0.704-0.996	0.048

SR: sinus rhythm; AF: atrial fibrillation; CI: confidence interval; HR: heart rate; HHR1: heart rate recovery in the first minute after finishing CPET; SBP: systolic blood pressure; CPET: cardiopulmonary exercise test.

HFSS, Sodium, NT-proBNP, right ventricular dysfunction, LVEF, CPET duration, heart rate recovery in the first minute after finishing CPET (HHR1) and initial and maximal SBP during CPET were predictors of the primary endpoint in both groups.

With the exception of HHR1, heart rate (HR) parameters during CPET were only predictors of the primary endpoint in the AF group, as seen with lower values of maximal HR, lower values of maximal (%) predicted HR and a lower variation of the HR during exercise, for patients with AF for whom the primary endpoint occurred and for those for whom it did not, respectively (Table 4).

On the other hand, the use of BB was only a predictor of the primary endpoint in the SR group (Table 3).

### Relationship between CPET prognostic parameters and primary outcome

The power to predict the primary outcome by CPET parameters is represented in the supplementary index. Univariate Cox analysis shows that pVO_2_, pVO_2_ (%) predicted, pVO_2_ at AT, VE/VCO_2_ slope and OUES are all predictors of the primary outcome in both groups (p < 0.05 for all).

In addition to the Cox analysis, these CPET parameters were analysed for the highest AUC in the 12 months’ follow-up period. In the SR group, VE/VCO_2_ slope had the highest AUC value (0.906) followed by predicted pVO_2_ (%) (0.903), with OUES with the lower AUC value (0.798). Despite these numerical differences, no statistically significant difference was found when the Hanley & McNeil test was applied to compare the different AUC values of the CPET parameters.

In the AF group, predicted pVO_2_ (%) (0.878) and pVO_2_ (0.869) had the highest AUC values. Similarly to the SR group, OUES had the lowest AUC value (0.833), but no statistically significant difference was found when the Hanley & McNeil test was applied to compare these parameters.

The Hanley & McNeil test was applied for comparing each CPET AUC parameter in the AF versus SR groups as well, with no statistically significant difference found.

Multivariate Cox analysis (Table 5) showed that when pVO_2_ and the VE/VCO_2_ slope are analysed together, significant differences were found between SR and AF groups. In the SR group, pVO_2_ lost his predictive power (p = 0.280) while the VE/VCO_2_ slope remained predictive of the primary outcome (p = 0.001). In the AF group, the VE/VCO_2_ slope lost its predictive power (p = 0.398) and pVO_2_ showed a trend towards the prediction of the primary outcome (p = 0.091).

**Table 5:** Multivariate Cox analysis of CPET^1^ prognostic parameters

Multivariate Cox analysis	SR			AF		
	Hazard ratio	95% CI	p	Hazard ratio	95% CI	p
1) pVO_2_ vs VE/VCO_2_ slope						
pVO_2_	0.910	0.766-1.080	0.280	0.759	0.551-1.045	0.091
VE/VCO_2_ slope	1.117	1.045-1.194	0.001	1.050	0.937-1.177	0.398
2) pVO_2_ (%) predicted vs VE/VCO_2_ slope						
pVO_2_ (%)	0.933	0.888-0.981	0.006	0.942	0.879-1.010	0.094
VE/VCO_2_ slope	1.070	1.005-1.139	0.033	1.078	0.974-1.193	0.145
3) OUES^5^ vs VE/VCO_2_ slope						
OUES	1.508	0.388-5.864	0.553	0.624	0.056-6.975	0.701
VE/VCO_2_ slope	1.170	1.090-1.256	< 0.001	1.123	1.002-1.258	0.046
4) pVO_2_ vs. OUES						
pVO_2_	0.742	0.597-0.922	0.007	0.623	0.482-0.907	0.014
OUES	1.061	0.183-6.153	0.948	2.335	0.156-34.907	0.539

SR: sinus rhythm; AF: atrial fibrillation; CPET: cardiopulmonary exercise test; CI: confidence interval; pVO2: peak O2 consumption; OUES: oxygen uptake efficiency slope.

Similar results were found in the multivariate Cox analysis of predicted pVO_2_ (%) and the VE/VCO_2_ slope in the AF group (p = 0.094 and p = 0.145, respectively), while in the SR group there was a difference, since predicted (%) pVO_2_ (p = 0.006) and VE/VCO_2_ slope (p = 0.033) kept their predictive power (p = 0.006), while pVO_2_ had not (p = 0.280).

OUES lost its predictive power in the multivariate Cox analysis in both SR and AF groups when compared with pVO_2_ (p = 0.948 and p = 0.539, for SR and AF group respectively) and when compared with the VE/VCO_2_ slope (p = 0.503 and p = 0.701, for SR and AF group respectively).

### Cut-off value for HT selection: PPV and NPV for the primary outcome

The univariate Cox analysis for the primary outcome of the two recommended CPET cut-offs for HT selection^7^ (pVO_2_ ≤ 12 ml/kg/min or ≤ 14 ml/kg/min without BB and VE/VCO_2_ slope ≤ 35) is represented in Table 6, showing that in the two groups, both cut-offs remained predictors of the outcome.

**Table 6:** Univariate Cox analysis for the primary outcome of the two recommended cardiopulmonary exercise test cut-offs for Heart Transplantation selection

	SR			AF		
	Hazard ratio	95% CI	p	Hazard ratio	95% CI	p
pVO_2_ ≤ 12 ml/kg/min	8.673	3.048-24.680	< 0.001	44.220	8.686-225.129	< 0.001
VE/VCO_2_ slope > 35	20.858	5.985-72.696	< 0.001	5.613	1.164-27.059	0.032

SR: sinus rhythm; AF: atrial fibrillation; CI: confidence interval; pVO_2_: peak O_2_ consumption.

In pVO_2_ ≤ 12 ml/kg/min or ≤ 14 ml/kg/min without BB, the PPV for the primary outcome was 100% in the AF group and 38.5% in the SR group (Table 7), with a NPV of 95.5% and 94.3% in the AF and SR groups, respectively. Higher values were found when the analysis excluded patients not doing BB, with a PPV of 100% and 75%, and a NPV of 97.1% and 95.3% for the AF and RS groups respectively.

**Table 7:** Proportion of patients correctely classified at 12 months of follow up

	AF	SR
pVO_2_ ≤ 12 ml/kg/min or ≤ 14 ml/kg/min without BB2	7/7 - 100%	5/13 - 38.5%
pVO_2_ > 12 ml/kg/min or > 14 ml/kg/min without BB	42/44 - 95.5%	198/210 - 94.3%
pVO_2_ ≤ 12 ml/kg/min only in patients doing BB	5/5 - 100%	6/8 - 75%
pVO_2_ > 12 ml/kg/min only in patients doing BB	34/35 - 97.1%	161/169 - 95.3%
VE/VCO_2_ slope > 35	7/21 - 33.3%	14/47 - 29.8%
VE/VCO_2_ slope ≤ 35	28/30 - 92.3%	173/176 - 98.3%

SR: sinus rhythm; AF: atrial fibrillation; pVO_2_: peak O_2_ consumption; BB: beta-blockers.

In VE/VCO_2_ slope > 35 (Table 7), lower values of PPV were reported (33.3% and 29.8% for AF and SR groups, respectively), with similar NPV to pVO_2_ (92.3% and 98.3% for AF and SR groups, respectively).

## Discussion

The presence of AF is associated with a negative prognostic effect in HF, with 50-90% increased mortality and HF progression in the Framingham Heart Study.^12^ Our population revealed some baseline differences between SR and AF groups, with some of that in previously described prognostic markers of HF, as AF patients were older,^13,14^ with lower GFR,^15-17^ with worse right ventricular function^18^ and a lower LVEF.^19,20^ In regard to CPET parameters, our AF patients revealed a lower exercise capacity than SR patients since they had a higher VE/VCO_2_ slope and a lower CPET duration, pVO_2_, OUES, time to AT and pVO_2_ at AT. As expected, these differences converted in a worse prognosis in the AF group, with a 2-fold increase in the primary endpoint events (17.6% VS 8.1%, p = 0.038) and 3-fold increase in all-cause mortality (17.6% VS 6.3%, p = 0.008) in the 1-year follow-up.

The majority of the predictors of the primary endpoint were predictors for both SR and AF groups. The HFSS,^21^ Sodium,^22^ NT-proBNP,^23-25^ right ventricular dysfunction,^18^ lower LVEF,^19,20^ CPET duration, HHR1,^26^ and initial and maximal SBP during CPET^27^ were included in this group, with all of them being formerly described as prognostic markers in HF patients.

Differences were found regarding maximal HR and variation of HR during the exercise, with lower values in AF patients predicting the primary outcome only in that group.

Patients not using BB were solely predictive of the primary outcome in the SR group, but not in the AF group. Whether this is in agreement with other studies that failed to reveal prognostic benefit from BB in the AF group of HF patients^28-30^ or to a underpowered analysis since only 11 patients in the AF group were not doing BB cannot be guaranteed.

### Cut-off value for HT selection: PPV and NPV for the primary outcome

**Figure 1:**
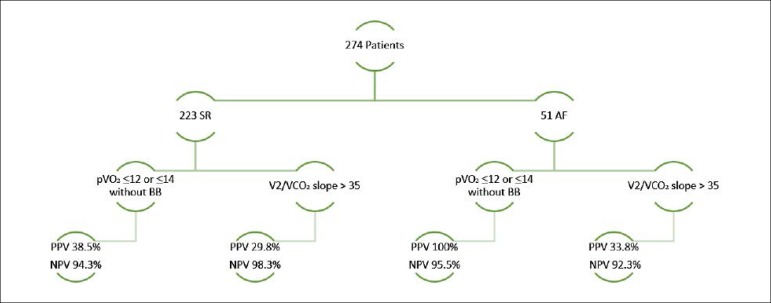
Positive (PPV) and negative predictive value (NPV) of pVO_2_ and VE/VCO_2_ slope.

Whether HF patients with AF can be precisely stratified with the current CPET cut-offs for HT selection have not been specifically studied before. The cut-off value for pVO_2_ showed a PPV for the primary outcome of 100% in the AF group and 38.5% in the SR group, with a NPV of 95.5% and 94.3% in the AF and SR groups, respectively. Hence, despite AF carries a worse prognosis in HF patients, the current cut-off of pVO_2_ for HT selection can precisely stratified these high-risk patients, with no patients under the cut-off misdiagnosed as high risk patients and less than 5% of patients above the cut-off having the primary outcome in the 1-year follow-up (Figure 1). These results suggest that patients under the cut-off of pVO_2_ should be managed accordingly, considering quickly referring for HT or mechanical circulatory support, since medical treatment is associated with negative outcomes in a 1-year period, and that we can be relatively safe in regard to 1-year outcomes of patients above the cut-off.

In regard to SR patients, the lower risks associated are responsible for a lower value of PPV above the pVO_2_ cutoff. The PPV was raised from 38,5% to 75% when the analysis excluded patients not doing BB. The NPV remains high in this group (94,3%).

During exercise, both CO_2_ output and ventilation increase steadily, but in patients with HF, the slope of the relationship is increased.^31^ Previous studies have confirmed the prognostic impact of VE/VCO_2_ in patients with HF, with higher values being associated with worse outcomes.^32-35^ However, the value of VE/VCO_2_ in AF patients with HF is not so well established, with differences in results in some trials.^36,37^

In our study, with a VE/VCO_2_ slope > 35, lower values of PPV were reported (33.3% and 29.8% for AF and SR groups, respectively), with similar NPV compared to pVO_2_ results (92.3% and 98.3% for AF and SR groups, respectively, figure 1). The power to predict the primary outcome by the VE/VCO_2_ slope, revealed an AUC of 0.906 for the SR group (the highest of all the CPET parameters analysed) and 0.844 in the AF group, with no statistically significant difference found when comparing the different AUC values of the CPET parameters. These differences in PPV may suggest that despite the fact that VE/VCO_2_ slope could be at least as good for prognostic assessment in HF patients as pVO_2_, the cut-off to use with the VE/VCO_2_ slope is not so well established as the cut-off for pVO_2_ in AF patients.

One previous study has shown that in a multivariate Cox analysis, pVO_2_ was identified as a sole significant predictor of cardiac events in HF patients in SR and the VE/VCO_2_ slope in AF patients.^38^ Our results, however, do not concur with the previous results. In fact, our multivariate Cox analysis (Table 5) showed that when pVO_2_ and the VE/VCO_2_ slope are analysed together, pVO_2_ lost its predictive power (p = 0.280) while the VE/VCO_2_ slope remained predictive of the primary outcome (p = 0.001) in the SR group. In the AF group, the VE/VCO_2_ slope lost its predictive power (p = 0.398) while pVO_2_ showed a trend for the prediction of the primary outcome (p = 0.091).

The predicted pVO_2_ (%) has been demonstrated as a useful prognostic marker in previous HF studies.^39^ In the multivariate Cox analysis of predicted pVO_2_ (%) and the VE/VCO_2_ slope, predicted pVO_2_ (%) kept his predictive power in the SR group (p = 0.006) in contrast to pVO_2_, while in the AF group, it showed a trend towards prediction of the primary outcome (p = 0.094) and had the highest AUC predictive value (0.878).

OUES is derived by plotting VO_2_ as a function of log10VE, which is an approximately linear relation, indicating how effectively O_2_ is extracted and taken into the body.^40^ In HF patients, OUES is reduced in proportion to disease severity and linked to outcome.^41,42^ In our population, OUES had the numerically lower AUC for predicting the primary outcome in both AF and SR groups and lost its predictive power in the multivariate Cox analysis when compared with pVO_2_ and when compared with the VE/VCO_2_ slope, which is in accordance with other previous study.^43^

### Study limitations

There are limitations to our study that should be referenced. Even though data was obtained from the outpatient clinic visits, medical charts were reviewed and complemented with a standardized telephone interview to all patients at 12 months of follow-up to collect data for the primary and secondary outcomes. Information pertaining to the selection or not of rhythm control for the treatment of AF was not gathered. Despite this, the goal of the trial was to define, during the initial evaluation, which patients needed early indication for HT or mechanical circulatory support, reducing the importance of the aforementioned information.

Despite being a seven-year follow-up of patients evaluated for HT in one advanced HF centre, the analysed cohort was not larger than other studies of the relation between HF and AF.^2,36,38^ However, the sample size is similar to other studies that highlighted the value of CPET parameters, including for the selection of patients for HT.^8,32,35,44,45^

Since patients were referred for a tertiary hospital for the purpose of evaluation with HF team and possible indication for HT or mechanical circulatory support, these patients may not be representative of the older or with higher comorbidities HF community, who are not candidate for advanced HF treatment.

## Conclusions

Despite AF carries a worse prognosis for the HF patients, the current cut-off of pVO_2_ for HT selection can precisely stratify this group of high-risk patients. The findings from the present study suggest that HF patients with AF and a CPET under the current cut-off of pVO_2_ for HT selection should be quickly referred for HT or mechanical circulatory support, since medical treatment is associated with negative outcomes in a 1-year period, with a higher PPV than patients in SR. In addition, pVO_2_ cut-off seems to have higher PPV than VE/VCO_2_ slope cut-off for the prediction of the primary outcome in HF patients with AF.
